# Obesity and weight loss differentially regulate autophagy in mouse ovarian granulosa cells

**DOI:** 10.5935/1518-0557.20260039

**Published:** 2026

**Authors:** Feng Li, Xinyan Chen, Xi Zhang, Jiajia Lu, Chenchen Xu, Huiming Ju, Tongmin Xue

**Affiliations:** 1 Reproductive Medicine Center, Northern Jiangsu People’s Hospital, Yangzhou 225009, China; 2 Jiangsu Co-innovation Center for Prevention and Control of Important Animal Diseases and Zoonoses. College of Veterinary Medicine, Yangzhou University, Yangzhou 225009, China; 3 Northern Jiangsu People’s Hospital affiliated with Yangzhou University, Yangzhou 225009, China

**Keywords:** obesity, weight loss, granulosa cell, autophagy

## Abstract

**Objective:**

This study aimed to elucidate the impact of obesity and weight loss on autophagic processes in ovarian granulosa cells (GCs), given the crucial roles that these cells and this process play in oogenesis.

**Methods:**

Three experimental groups were used: a control group (ND group), a high-fat diet-induced obese mouse model group (HFD group), and a swimming exercise group (SE group). Immunohistochemistry (IHC) and Western Blotting (WB) were used to detect protein expression in GCs.

**Results:**

LC3B expression was higher in the HFD and SE groups than in the ND group (*p*<0.01), but lower in the SE group than in the HFD group (*p*<0.05). p62 expression was lower in the HFD and SE groups than in the ND group (*p*<0.01 and *p*<0.05, respectively), but higher in the SE group than in the HFD group (*p*<0.05). The LC3B-II/I ratio was elevated in the HFD group versus the ND and SE groups (*p*<0.05). p62 expression was lower in the HFD group than in the SE and ND groups (*p*<0.05 and *p*<0.01, respectively), and lower in the SE group than in the ND group (*p*<0.01). In IHC, the p-mTOR/mTOR ratio was lower in the HFD group than in the ND group (*p*<0.05), but higher in the SE group than in the ND and HFD groups (*p*<0.01). The AMPK/AMPK ratio was higher in the HFD group than in the ND group (*p*<0.01), but lower in the SE group than in the HFD group (*p*<0.01). In WB, the p-mTOR/mTOR ratio was lower in the HFD and SE groups than in the ND group (*p*<0.01), but higher in the SE group than in the HFD group (*p*<0.05). p-RPS6/RPS6 ratio was lower in the HFD group than in the ND group (*p*<0.01), but higher in the SE group than in both groups (*p*<0.01).

**Conclusions:**

Our findings suggest that obesity activates granulosa cell autophagy *via* AMPK/mTOR signaling, while weight loss partially restores homeostasis.

## INTRODUCTION

Obesity is a prevalent chronic condition and a major contributor to global infertility. According to a 2016 Lancet report based on global body weight surveys, China has the highest number of obese individuals, with 43.2 million obese men and 46.4 million obese women (NCD Risk Factor Collaboration, 2016; Poston *et al*., 2016). Obesity impairs female reproductive function, leading to ovulatory disorders, reduced fertility, and poorer outcomes in assisted reproductive technologies (ART), along with increased risks of miscarriage, stillbirth, and preeclampsia (Kanninen *et al*., 2013). Although obesity has long been linked to infertility, the precise mechanisms remain unclear (Talmor & Dunphy, 2015).

Weight loss strategies include exercise, dietary interventions, and pharmacological treatments, with exercise being particularly beneficial due to its positive effects on weight reduction, lipid metabolism, and inflammation (Kirk *et al*., 2019). Our preliminary studies using obese female mouse models demonstrated that obesity impairs reproductive capacity, while combined exercise and dietary interventions significantly improve it (Yu *et al*., 2024).

Autophagy, a key cellular degradation and recycling pathway, is essential for maintaining homeostasis and reproductive development. Research indicates that autophagy regulates granulosa cell apoptosis in various species, including rats and humans (Gao *et al*., 2023). Ovarian GCs, a major ovarian cell population, support oocyte development by synthesizing progesterone and converting androgens into estrogens, thereby playing a critical role in folliculogenesis and maturation (Richards & Pangas, 2010; Yang *et al*., 2022).

Obesity-related metabolic complications are often associated with autophagy dysregulation, as autophagy monitors cellular energy balance during nutrient deprivation. Disrupted autophagy due to dyslipidemia or overnutrition can lead to metabolic disorders (Zhang *et al*., 2018). However, the role of autophagy in obesity-induced reproductive dysfunction remains poorly understood. This study investigates how obesity and subsequent weight loss affect autophagy in ovarian GCs, aiming to elucidate the potential mechanisms of obesity-related ovarian dysfunction and provide theoretical insights for improving female fertility. We hypothesized that obesity induces excessive autophagy in ovarian granulosa cells via AMPK/mTOR dysregulation and that weight loss reverses these alterations.

## MATERIALS AND METHODS

### Experimental Animals and Experimental Methods

Female C57BL/6J mice were obtained from the Comparative Medical Experimental Animal Center of Yangzhou University.

### Establishment of Obese and Weight-Loss Mouse Models

Three-week-old female C57BL/6J mice were randomly assigned to groups (n=20): Normal diet (ND) group: fed a standard diet; High-fat diet (HFD) group: fed a 60% kJ HFD for 16 weeks to induce obesity (obesity was confirmed when body weight was ≥30% higher than that of the ND group); Swimming exercise (SE) group: obese mice (from the HFD group) were switched to standard chow and subjected to swimming exercise to induce weight loss (50 min/day, 6 days/week) (Yu *et al*., 2024). All mice were maintained under SPF conditions (12-h light/dark cycle, ad libitum access to water) at Yangzhou University’s Experimental Animal Center. During the experiments, the standard diet (10% kJ, #2109513) and high-fat diet (HFD, 60% kJ, #2109510) were purchased from Jiangsu Medison Biopharmaceutical Co., Ltd.

For the detection of GC autophagy, polyacrylamide gel electrophoresis was used to detect autophagy-related protein expression. β-actin (sc-48166, Santa Cruz, USA) was used as internal reference protein. The following autophagy-related proteins were detected: LC3B (Ab229327, Abcam, UK), P62 (18420-1-AP, Proteintech, USA), phospho-mTOR (p-mTOR, 6778-1-1g, Proteintech, USA), phospho-RPS6 (p-RPS6, HA721589, HUABIO, China), RPS6 (HA600084, HUABIO, China), phospho-AMPKα (p-AMPKα, IPH0009, Baijia Biotechnology, China), AMPKα (2532, Cell Signaling Technology, USA), HRP-conjugated goat anti-rabbit IgG (sc-2004, Santa Cruz, USA), and HRP-conjugated goat anti-mouse IgG (ASP1613, Abcepta, China).

### Isolation of Ovarian GCs

Mice were injected intraperitoneally with 10 IU PMSG and euthanized 48 h later. Ovaries were collected and rinsed in DMEM/F12 medium containing 3% FBS. GCs were isolated by follicular puncture under a stereomicroscope.

### Protein Extraction and Quantification

GCs were lysed in RIPA buffer supplemented with PMSF and phosphatase inhibitors, followed by sonication (on ice) and centrifugation. Protein concentration was determined using the BCA assay.

### Western Blot (WB) and Immunohistochemistry (IHC)

**WB:** Proteins were separated by SDS-PAGE and transferred to PVDF membranes. After blocking (2% BSA in PBST, 1 h, RT), the membranes were incubated with primary antibodies (4°C, overnight) and HRP-conjugated secondary antibodies (RT, 2 h). Chemiluminescence signals were captured and analyzed using ImageJ.

**IHC:** Ovarian tissues were fixed in 4% paraformaldehyde, paraffin-embedded, and sectioned (5µm). After deparaffinization, the sections were blocked (5% BSA, 30 min), incubated with primary antibodies (4°C, overnight) and secondary antibodies, and developed with DAB. Image analysis was performed using ImageJ.

### Statistical Analysis

Data are presented as x±SD. All data collection and image analyses were performed by investigators blinded to group allocation, and statistical analyses were performed using GraphPad Prism 7 (GraphPad Software, La Jolla, CA, USA). One-way ANOVA was used to analyze differences in protein levels. A correction for multiple comparisons (e.g., Tukey’s test) was applied where appropriate. Significance thresholds were set *p<0.05 and **p<0.01.

## RESULTS

### IHC Analysis of LC3B and p62

**Figure 1:**
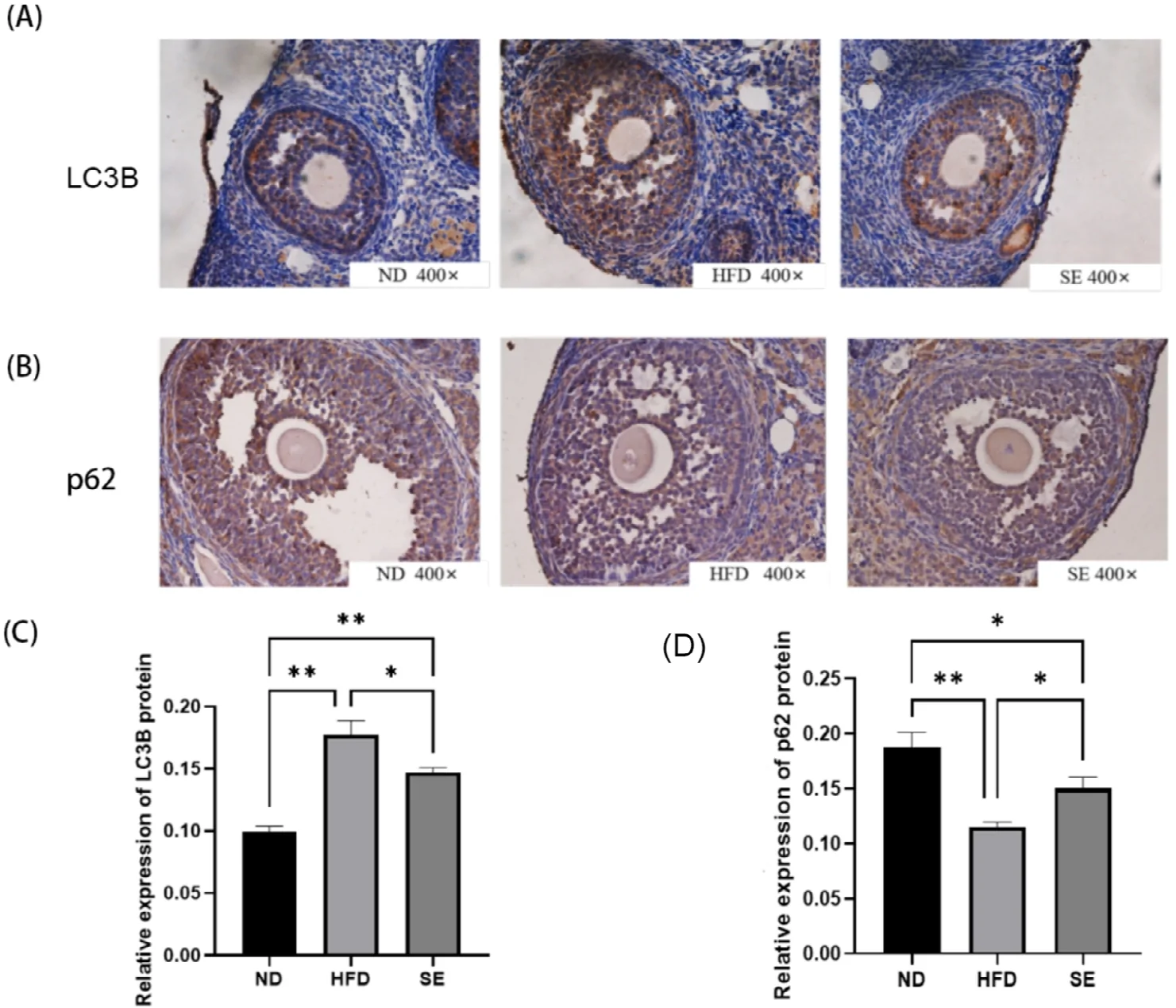
Immunohistochemical detection of LC3B and p62 expression in mouse ovarian tissues from ND, HFD, and SE groups

IHC staining results indicated that the mean expression levels of LC3B in the ND, HFD, and SE groups were 0.10±0.01, 0.18±0.01, and 0.15±0.01, respectively. Correspondingly, the mean expression levels of p62 in these groups were 0.19±0.04, 0.12±0.01, and 0.15±0.03 (Figure 1). The immunohistochemistry (IHC) results demonstrated that LC3B expression was significantly higher in both the HFD and SE groups compared to the ND group both (*p*<0.01), though the SE group exhibited lower LC3B levels than the HFD group (*p*<0.05). Conversely, p62 expression was significantly reduced in both the HFD group (*vs*. ND, *p*<0.01) and the SE group (*vs*. ND, *p*<0.05), yet remained higher in the SE group than in the HFD group (*p*<0.05).

(A) Representative LC3B staining in ovarian sections (ND, n=5; HFD, n=6; SE, n=7).

(B) Representative p62 staining in ovarian sections (ND, n=7; HFD, n=10; SE, n=8).

(C) Quantitative analysis of LC3B expression.

(D) Quantitative analysis of p62 expression.

Data are expressed as mean ± SEM. *p<0.05, **p<0.01.

### WB Analysis of LC3B and p62

**Figure 2:**
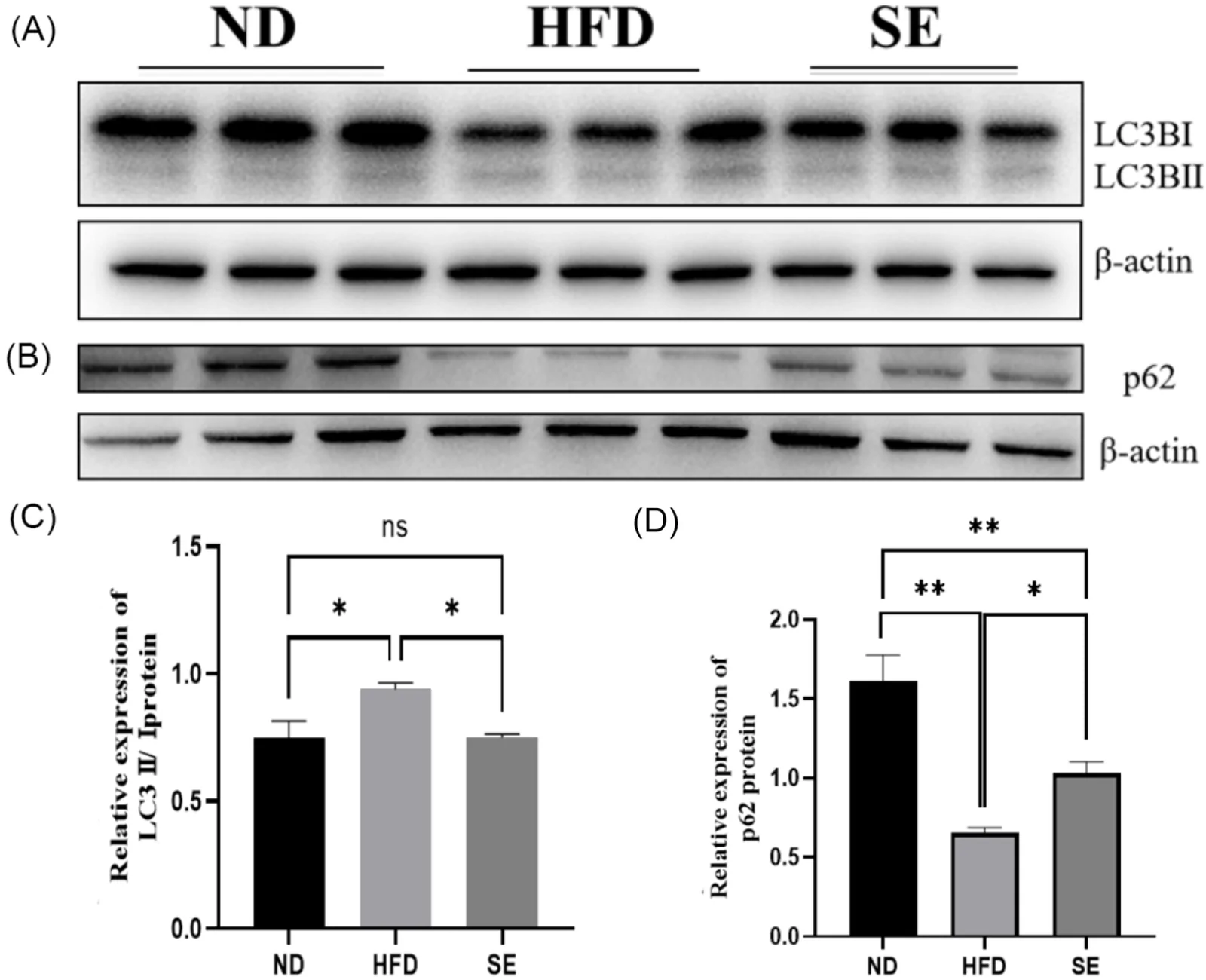
Western blot analysis of LC3B and p62 expression in ovarian granulosa cells from ND, HFD, and SE groups

WB results (Figure 2) revealed the following findings: the LC3B-II/I ratios in the ND, HFD, and SE groups were 0.75±0.16,.94±0.06, and 75±0.03, respectively. Regarding p62 expression, the mean levels were 1.61±0.38 in the ND group, 0.65±0.08 in the HFD group, and 1.03±0.17 in the SE group.

(A) Representative Western blot bands of LC3B-I and LC3B-II in GCs (ND, n=6; HFD, n=6; SE, n=6).

(B) Representative Western blot bands of p62 in GCs (ND, n=5; HFD, n=6; SE, n=6).

(C) Grayscale quantification of LC3B-II/I ratio.

(D) Grayscale quantification of p62 expression level.

Data are expressed as mean ± SEM. Significant differences are indicated as follows: *p<0.05, **p<0.01

Western blot (WB) analysis revealed that the LC3B-II/I ratio was significantly elevated in the HFD group relative to both the ND and SE groups (both *p*<0.05). For p62, expression was significantly lower in the HFD group than in both the SE group (*p*<0.05) and the ND group (*p*<0.01), while the SE group also showed lower p62 levels than the ND group (*p*<0.01).

### Immunohistochemical Analysis of p-mTOR/mTOR and p-AMPK/AMPK

**Figure 3:**
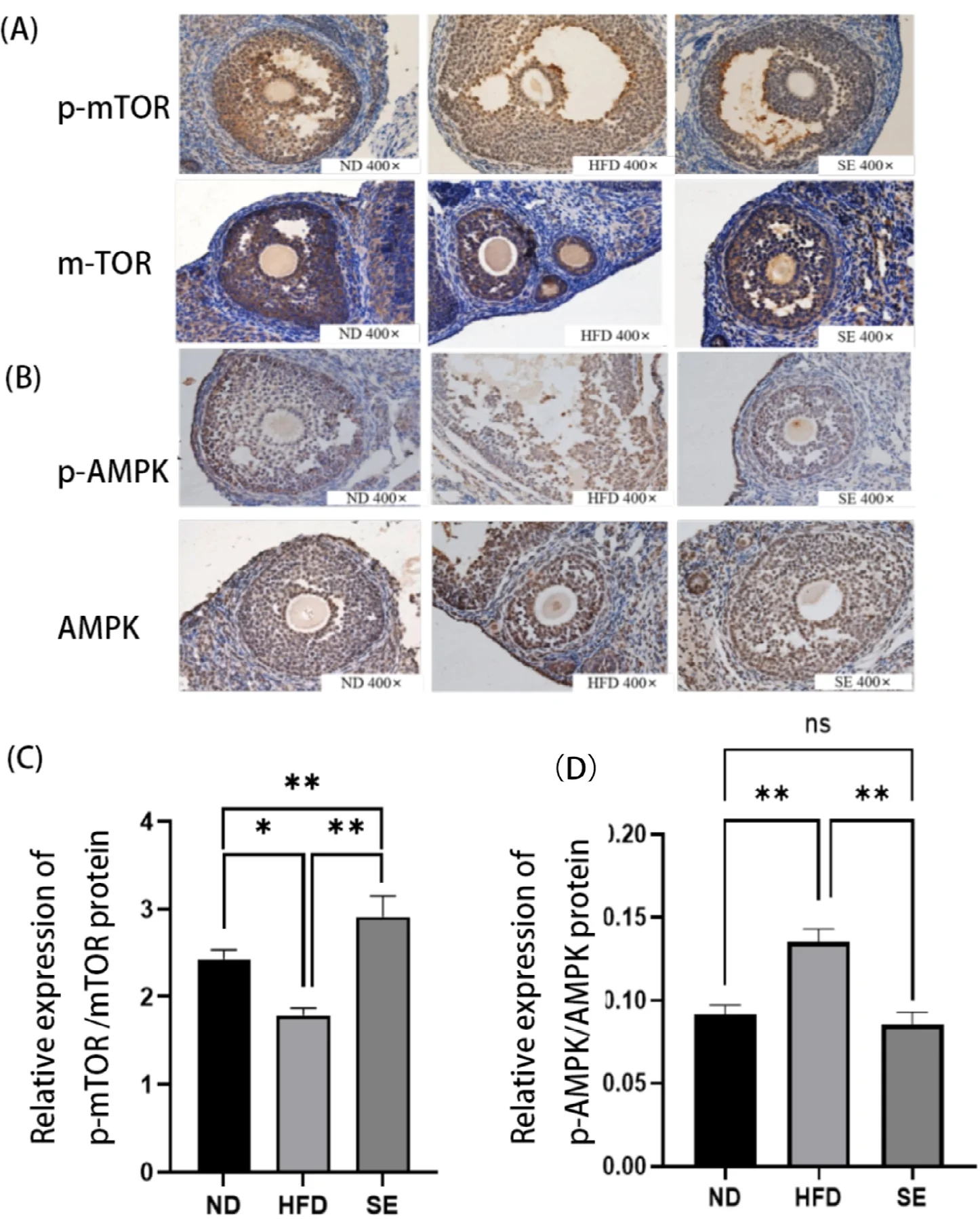
Immunohistochemical analysis of p-mTOR/mTOR and p-AMPK/AMPK expression in ovarian granulosa cells under obesity and exercise-induced weight loss conditions

IHC data (Figure 3) demonstrated that the p-mTOR/mTOR ratios in the ND, HFD, and SE groups were 2.43±0.30, 1.79±0.22, and 2.91±0.67, respectively. Conversely, the p-AMPK/AMPK ratios in the ND, HFD, and SE groups were 0.09±0.01, 0.14±0.02, and 0.09±0.02, respectively.

(A) Representative immunohistochemical staining of p-mTOR/mTOR in ovarian paraffin sections (ND, n=8; HFD, n=8; SE, n=8).

(B) Representative immunohistochemical staining of p-AMPK/AMPK in ovarian paraffin sections (ND, n=6; HFD, n=9; SE, n=8).

(C) Quantitative analysis of p-mTOR/mTOR expression levels.

(D) Quantitative analysis of p-AMPK/AMPK expression levels.

Data are expressed as mean ± SEM. Statistical significance is indicated as follows: *p<0.05, **p<0.01.

IHC further indicated that the p-mTOR/mTOR ratio was significantly lower in the HFD group than in the ND group (*p*<0.05), but markedly higher in the SE group than in both the ND and HFD groups (both *p*<0.01). The p-AMPK/AMPK ratio was significantly increased in the HFD group relative to the ND group (*p*<0.01), whereas it was reduced in the SE group compared with the HFD group (*p*<0.01), with no significant difference between the ND and SE groups (*p*>0.05).

These findings suggest that obesity may modulate granulosa cell autophagy through the AMPK/mTOR signaling pathway.

### WB Analysis of p-mTOR/mTOR and p-RPS6/RPS6

**Figure 4:**
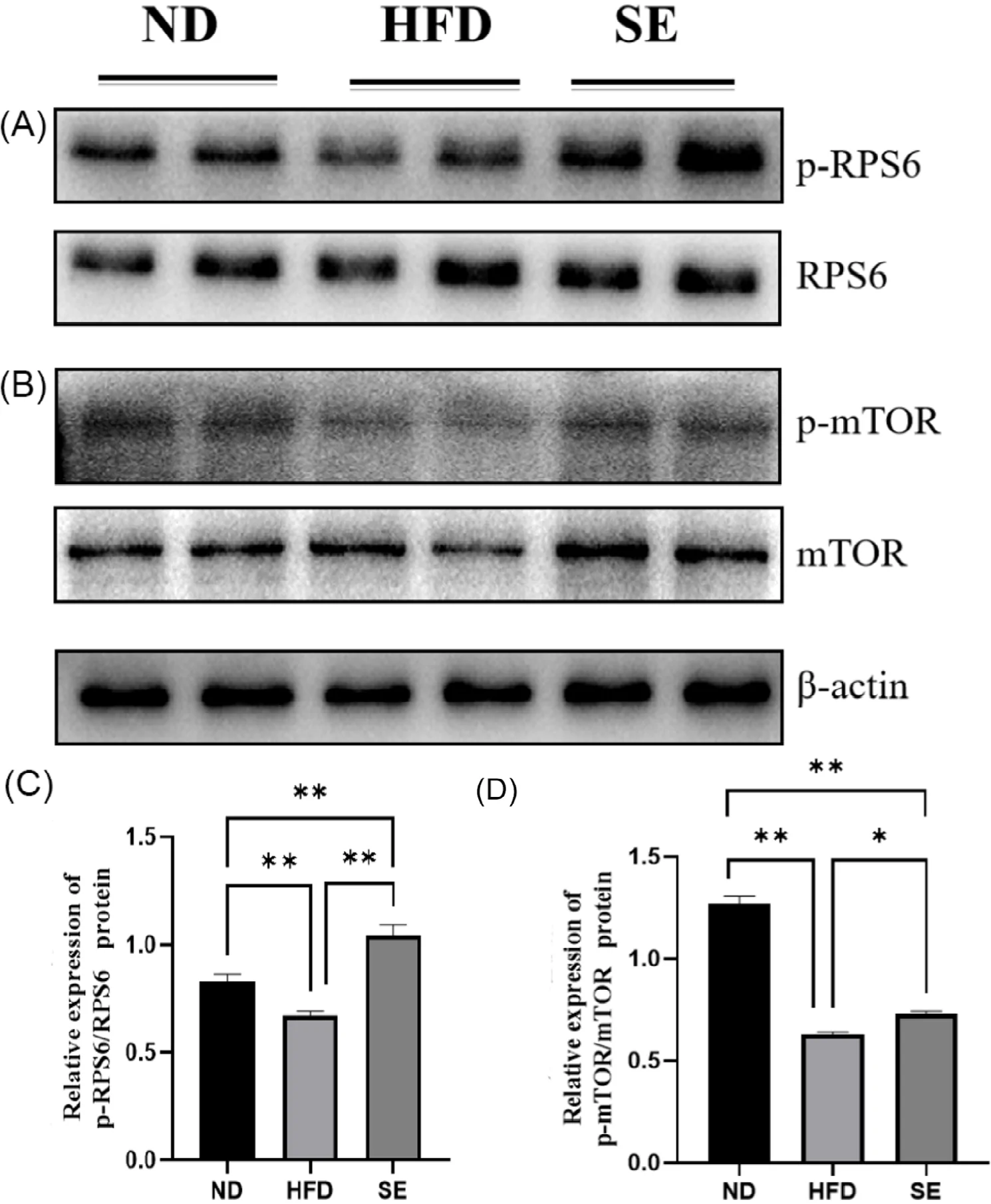
Western blot analysis of p-mTOR/mTOR and p-RPS6/RPS6 expression in ovarian granulosa cells under obesity and exercise-induced weight loss conditions

The p-mTOR/mTOR ratios in the HFD, ND, and SE groups were 0.63±0.03, 1.27±0.10, and 0.73±0.03, respectively. Correspondingly, the p-RPS6/RPS6 ratios in these groups were 0.67±0.05, 0.83±0.08, and 1.05±0.13 (Figure 4).

(A) Representative Western blot bands of p-mTOR/mTOR in GCs (ND, n=7; HFD, n=7; SE, n=7).

(B) Representative Western blot bands of p-RPS6/RPS6 in GCs (ND, n=7; HFD, n=7; SE, n=7).

(C) Grayscale quantification of p-mTOR/mTOR expression ratio.

(D) Grayscale quantification of p-RPS6/RPS6 expression ratio.

Data are expressed as mean ± SEM. Statistical significance is indicated as follows: *p<0.05, **p<0.01.

Regarding downstream mTOR signaling, both the p-mTOR/mTOR and p-RPS6/RPS6 ratios were significantly lower in the HFD group than in the ND group (both *p*<0.01). In the SE group, the p-mTOR/mTOR ratio was lower than that in the ND group (*p*<0.01) but higher than that in the HFD group (*p*<0.05). In contrast, the p-RPS6/RPS6 ratio in the SE group was significantly elevated compared with both the HFD and ND groups (both *p*<0.01).

These findings further support the involvement of the mTOR pathway in obesity-induced autophagy enhancement.

## DISCUSSION

Obesity has become a global health epidemic, with the World Health Organization estimating that over 300 million individuals are affected worldwide (NCD Risk Factor Collaboration, 2016; Poston *et al*., 2016; Gao *et al*., 2019). Our preliminary studies established that obesity negatively affects reproductive outcomes in female mice, as characterized by reduced oocyte quantity, compromised oocyte quality, and impaired blastocyst formation. Importantly, these effects were reversible through weight-loss interventions, which significantly improved multiple fertility parameters (Wang *et al*., 2023; Yu *et al*., 2024).

Ovarian GCs play essential roles in reproductive physiology by providing nutritional support and hormonal regulation. These cells are primarily responsible for estrogen and progesterone production during early luteinization and are crucial for proper follicular development and maturation (Zhang *et al*., 2017; Gao *et al*., 2023). Autophagy, a conserved cellular degradation process, maintains cellular homeostasis by balancing macromolecule biosynthesis and catabolism. This process is physiologically significant throughout reproduction, participating in primordial follicle development, spermatogenesis, embryogenesis, and placental homeostasis (Kanninen *et al*., 2013). Therefore, examining obesityand weight loss-induced alterations in granulosa cell autophagy provides valuable insights into their reproductive consequences.

LC3B and p62 serve as established autophagy markers. LC3B exists in two forms: cytoplasmic LC3B-I and membrane-bound LC3B-II, with the formation of the latter indicating autophagosome maturation. Conversely, p62 accumulation typically reflects autophagy suppression (Yoshii & Mizushima, 2017; Wang *et al*., 2023). Our immunohistochemical and WB analyses revealed that obesity significantly enhanced autophagy in ovarian GCs, while combined exercise and dietary interventions partially restored autophagy levels.

The AMPK/mTOR pathway is a central regulator of cellular energy metabolism and autophagy (González *et al*., 2020; Han *et al*., 2022). AMPK-mediated autophagy activation occurs through inhibition of mTOR phosphorylation, whereas mTOR activation promotes anabolic processes via downstream effectors such as RPS6KB1, thereby driving cellular growth and proliferation (Jhanwar-Uniyal *et al*., 2019; Guo *et al*., 2022; Wang *et al*., 2023). Our findings demonstrate that obesity-induced autophagy in GCs involves increased p-AMPK/AMPK ratios alongside decreased p-mTOR/mTOR and p-RPS6/RPS6 ratios. Notably, we observed divergent trends in p-mTOR/mTOR ratios between the IHC and WB analyses, which may reflect methodological differences in detecting phosphorylation states or subcellular localization. Nevertheless, both methods support the conclusion that obesity dysregulates mTOR signaling and autophagy in GCs. Weight-loss interventions effectively modulated these changes.

While autophagy is essential for tissue homeostasis, our results suggest that obesity-induced autophagy may contribute to impaired granulosa cell function, a hypothesis that requires future validation through direct functional assays. This aligns with our previous findings in porcine skeletal muscle satellite cells, where moderate, short-term autophagy enhanced metabolic and differentiation capacities, whereas prolonged, severe autophagy exerted cytotoxic effects (Wang *et al*., 2023). The current study proposes that obesity elevates granulosa cell autophagy to potentially detrimental levels, while weight-loss interventions can mitigate these effects through autophagy normalization.

Although we identified significant alterations in autophagy pathways, the direct functional consequences for fertility (e.g., oocyte competence or ovulation efficiency) await further investigation. Furthermore, the contribution of stress to the observed effects in our exercise model needs to be clarified in future studies employing stress-controlled exercise regimens. Lastly, resolving the technical disparities between the IHC and WB findings will require more spatially resolved or single-cell analytical approaches to fully understand cell-specific autophagy regulation in the ovary.

## Declarations

Ethics approval: The experimental protocol was approved by the Experimental Animal Ethics Committee of Yangzhou University (YZU-lcyxy-n021).
